# Predictive Value of the Electrical Risk Score for Ventricular Arrhythmias in Heart Failure Patients with Primary Prevention Implantable Cardioverter Defibrillators

**DOI:** 10.3390/jcm15197588

**Published:** 2026-09-30

**Authors:** Pelin Aladag, Ilke Erbay, Naile Eris Gudul, Ahmet Avci

**Affiliations:** 1 Department of Cardiology, Bulent Ecevit University Faculty of Medicine, Zonguldak 67630, Turkey; ilkeerbay@gmail.com (I.E.); nailegudul@hotmail.com (N.E.G.); 2 Department of Cardiology, Kastamonu University Faculty of Medicine, Kastamonu 37150, Turkey; drahmetavci@yahoo.com

**Keywords:** heart failure, implantable cardioverter defibrillator shock, electrical risk score

## Abstract

**Background**: Implantable cardioverter defibrillators (ICDs) are essential for the primary prevention of sudden cardiac death in heart failure (HF); however, appropriate shocks increase mortality and impair quality of life. The electrical risk score (ERS) is an electrocardiographic (ECG)-based composite index with proven prognostic value, but its ability to predict ventricular arrhythmias in HF remains unclear. This study aims to evaluate the predictive value of the ERS for ventricular arrhythmia-related ICD shocks in HF patients undergoing primary prevention ICD implantation. **Methods**: In this study, 91 patients with HF were followed for 12 months after ICD implantation. The ERS was calculated from baseline ECGs. Patients were grouped based on ERS (<3 vs. ≥3), according to receiver operating characteristic curve analysis. Multivariate logistic regression was used to evaluate predictors of appropriate ICD shocks, and predictive performance was compared using the DeLong test. **Results**: Thirty-five patients (38.5%) experienced at least one appropriate ICD shock. The median ERS was significantly higher in the shock group compared to the non-shock group (*p* < 0.001). Patients with ERS ≥ 3 had significantly higher rates of the anti-tachycardia pacing therapy (*p* < 0.001) and HF-related hospitalization (*p* = 0.004). ERS was an independent predictor of ICD shocks (*p* < 0.001), with superior discriminative performance (AUC 0.882) compared to most ECG markers, including frontal QRS T angle, QRS duration, Tp-e interval, and heart rate. **Conclusions**: The ERS was associated with ventricular arrhythmia-related ICD shocks in HF patients undergoing primary prevention ICD implantation. The ERS may identify patients at increased arrhythmic risk and guides follow-up and ICD management.

## 1. Introduction

Sudden cardiac death remains a major cause of mortality in patients with heart failure (HF), primarily driven by malignant ventricular arrhythmias, despite advances in guideline-directed medical therapy [1]. Several landmark trials have demonstrated that implantable cardioverter defibrillators (ICDs) significantly reduce mortality in patients with left ventricular ejection fraction (LVEF) ≤ 35 and New York Heart Association (NYHA) class II or higher symptoms, leading to guideline recommendations supporting ICD implantation for primary prevention [2,3,4]. However, although appropriate ICD shocks are lifesaving, they have been shown to be associated with increased mortality risk and impaired quality of life [5,6,7]. Nevertheless, LVEF alone has limited ability to discriminate individual arrhythmic risk, and a considerable proportion of patients undergoing ICD implantation for primary prevention may not experience ventricular arrhythmias requiring device therapy during follow-up [8,9]. Therefore, identifying clinical and electrocardiographic (ECG) predictors may improve ventricular arrhythmic risk stratification and support individualized patient management [10,11].

According to the 2021 European Society of Cardiology HF guidelines, the 12-lead ECG is a fundamental tool in the diagnosis, risk stratification, and management of HF patients [4]. Previous studies have reported that various ECG parameters, including heart rate and markers of ventricular hypertrophy, depolarization, and repolarization, may predict adverse outcomes in this population [12,13,14]. However, these markers have often been evaluated individually, limiting their overall predictive value. To address this limitation, Aro et al. proposed the electrical risk score (ERS), a composite index that incorporates seven ECG markers representing different phases of the cardiac electrical cycle [15]. The ERS is a cumulative ECG-based score derived from seven simple ECG parameters. This score has demonstrated prognostic value in various cardiovascular populations [16,17,18].

To date, no study has specifically investigated the predictive value of the ERS for ventricular arrhythmia and appropriate ICD shocks in HF patients receiving ICD therapy for primary prevention. The aim of this study is to evaluate the prognostic value of the ERS in predicting appropriate ICD shocks during follow-up.

## 2. Materials and Methods

### 2.1. Study Design and Population

This retrospective observational study included patients with HF who underwent ICD implantation for primary prevention at Zonguldak Bülent Ecevit University between January 2022 and January 2025. Patients ≥ 18 years old, with LVEF ≤ 35% at the time of ICD implantation due to ischemic or non-ischemic cardiomyopathy, and with 12 months of follow-up after implantation were included. For study analyses, LVEF was derived from the echocardiographic assessment (Philips Healthcare, Amsterdam, The Netherlands) performed closest to the baseline ECG evaluation(GE Healthcare, Milwaukee, WI, USA). Demographic features, laboratory data, echocardiographic parameters, ECG findings, ICD therapies, and clinical outcomes were obtained from electronic medical records. Patients with complete bundle branch block, paced rhythm, advanced atrioventricular block, missing ECG data, inappropriate ICD shocks, or device malfunction were excluded.

### 2.2. ICD Control and Programming

**Figure 1:**
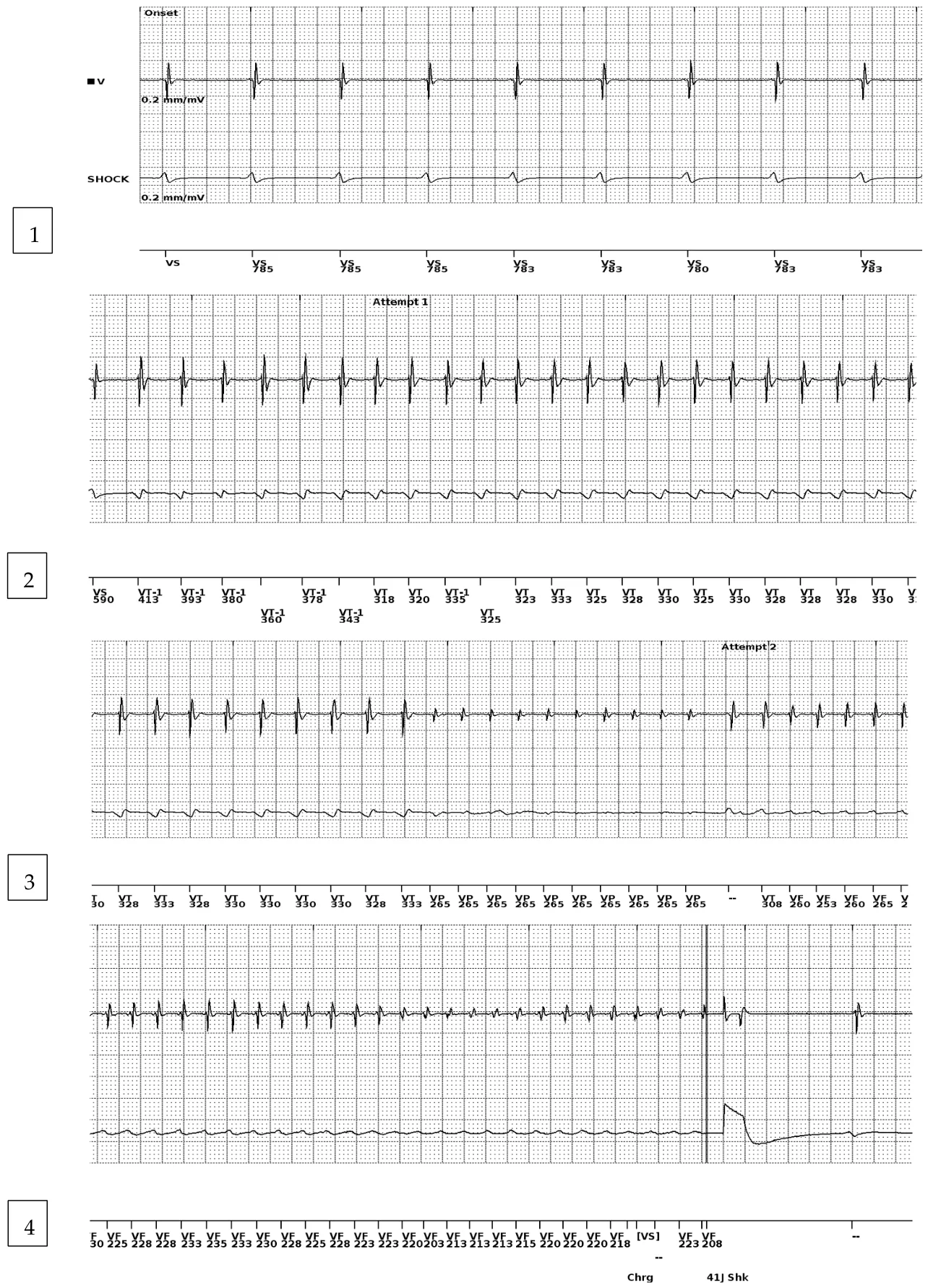
Device electrogram shape. (**1**) Basal rhythm. (**2**) Recognition of the onset of VT is displayed. (**3**) Anti-tachycardia pacing (ATP) therapy is practiced. (**4**) Defibrillation of the device with 41.0 j. and return to basal rhythm.

Patients were monitored for 12 months after ICD implantation at the pacemaker control outpatient clinic. Patients implanted with the device were evaluated monthly for the first 3 months and at subsequent 6-month intervals. All ICD controls were carried out under the supervision of a specialist doctor and an electrophysiology technician. In ICD programming, events with heart rates between 131 and 170 bpm were assigned to the monitoring zone. The ventricular tachycardia (VT) zone was set to 170 bpm followed by anti-tachycardia pacing with three bursts and ramps and if the arrhythmia persisted the ICD-applied defibrillator shock with 25 J and 35 J. The ventricular fibrillation (VF) zone was set to 207 bpm, followed by shock therapy at the maximum energy available according to device-specific programming (Figure 1). Only shocks delivered for documented VT or VF and confirmed by review of stored intracardiac electrograms were classified as appropriate ICD shocks.

### 2.3. Electrocardiographic Parameters

**Figure 2:**
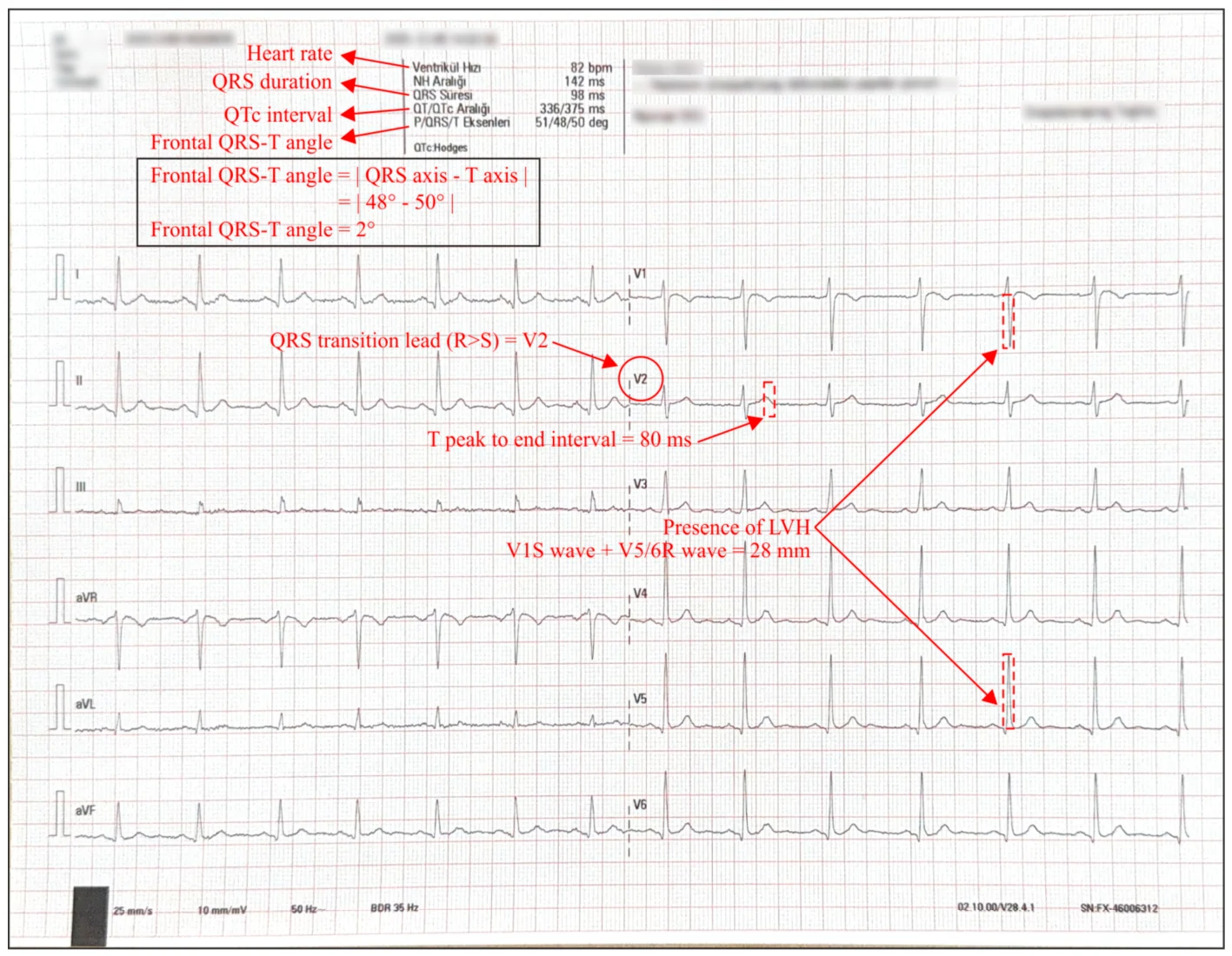
An example of the assessment of each component of the ERS from a 12-lead ECG.

Standard 12-lead resting ECGs were recorded at a paper speed of 25 mm/s and calibration of 10 mm/mV, and all tracings were evaluated by two independent cardiologists. The ERS was calculated according to the method described by Aro et al. [15] and consisted of seven ECG markers: heart rate, QRS duration, left ventricular hypertrophy (LVH) by Sokolow Lyon criteria, QTc interval, T peak to T end interval, QRS transition zone, and frontal QRS T angle (Figure 2). Each abnormal parameter was assigned one point. Abnormality thresholds were defined as follows: heart rate > 75 bpm, QRS duration > 110 ms, presence of LVH, QTc > 450 ms in men or >460 ms in women, Tp-e interval > 89 ms, transition zone at V4 or later, and frontal QRS T angle > 90 degrees. QTc values were corrected using the Bazett formula [15], and Tp-e measurements were obtained using the tangent method [19]. The frontal QRS T angle was calculated as the absolute difference between QRS and T axes, and values exceeding 180 degrees were recalculated as 360 minus the measured angle [16]. The LVH was defined as a voltage ≥ 35 mm (3.5 mV) according to the Sokolow–Lyon criteria by summing the voltage of the V1S wave + V5/6R wave on ECG [20]. The automatic ECG report indicated the presence of heart rate, QTc interval, QRS axis and T axis. Any discrepancies between observers were resolved by consensus.

### 2.4. Statistical Analysis

Data were analyzed using IBM SPSS Statistics for Windows, version 26 (IBM Corp., Armonk, NY, USA). Variables were tested for normality and presented as mean ± standard deviation or median (interquartile range), depending on distribution. Categorical variables were compared using the chi-square or Fisher’s exact test. Receiver operating characteristic (ROC) curve analysis was initially performed to evaluate the ability of the ERS to predict appropriate ICD shocks. The area under the curve (AUC) was calculated, and the optimal cut-off value for ERS was determined as 3 using the Youden index. Based on this cut-off, patients were divided into two groups: ERS < 3 and ERS ≥ 3. Univariable and multivariable logistic regression analyses were performed to evaluate factors associated with appropriate ICD shocks. The multivariable model included ERS and clinically relevant covariates, including age, LVEF, and N-terminal pro-B-type natriuretic peptide (NT-proBNP). NT-proBNP was expressed per 100 pg/mL increase for regression analysis. Odds ratios (ORs) and adjusted odds ratios (aORs) with 95% confidence intervals were reported. Pairwise comparisons of ROC curves were performed using the DeLong test implemented in MedCalc Statistical Software, version 20.106 (MedCalc Software Ltd., Ostend, Belgium). A two-sided *p* value < 0.05 was considered statistically significant.

## 3. Results

**Figure 3:**
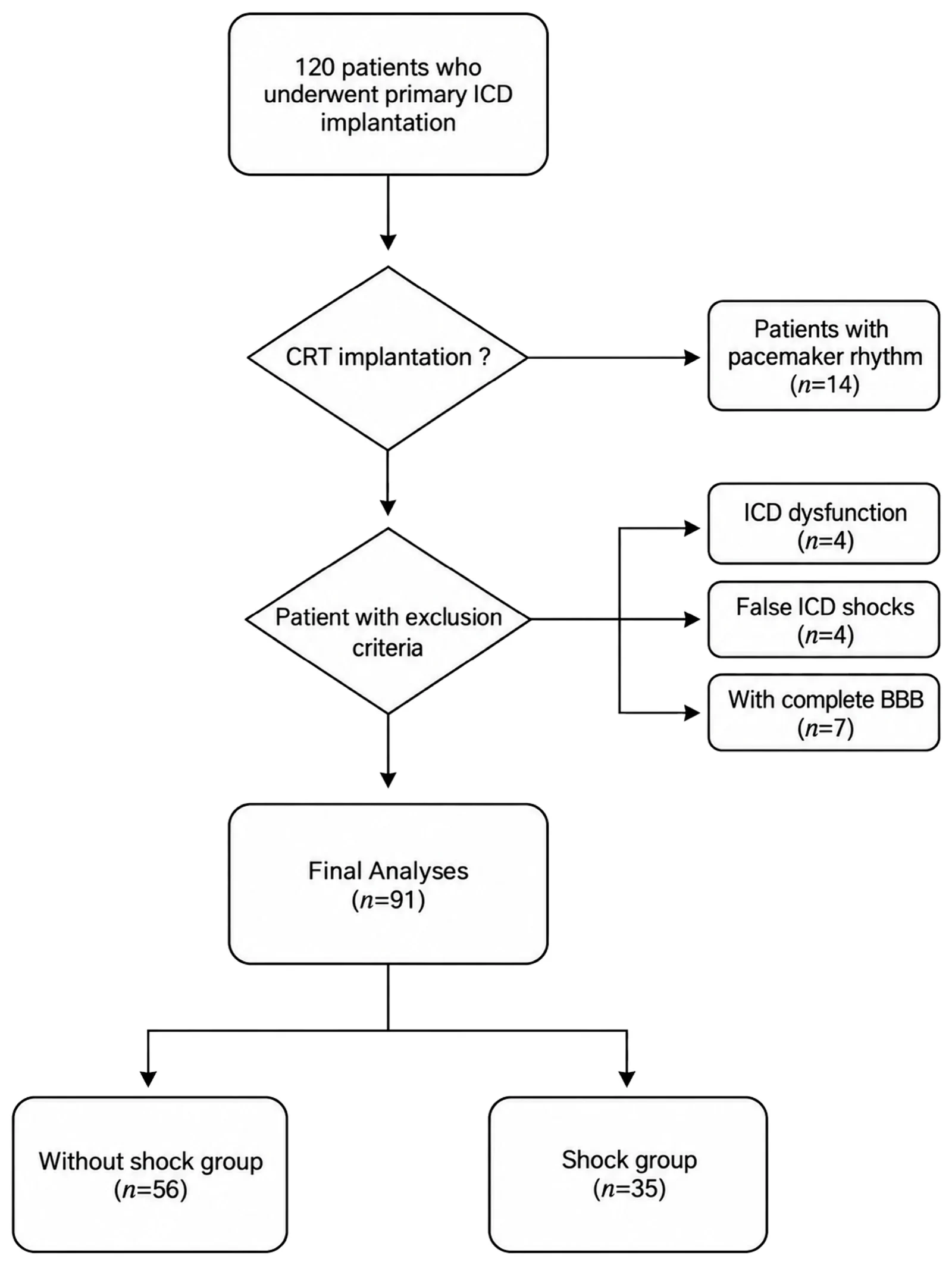
Study flowchart. BBB, bundle branch block; CRT, cardioverter resynchronization therapy; ICD, implantable cardioverter defibrillators.

A total of 120 patients who underwent ICD implantation for primary prevention were screened. Patients were excluded due to paced rhythm (*n* = 14), complete bundle branch block (n = 7), inappropriate ICD shocks (*n* = 4), and device malfunction (*n* = 4). After these exclusions, 91 patients were included in the final analysis. Of these, 56 patients did not experience ICD shocks, whereas 35 patients received at least one appropriate ICD shock during the 12-month follow-up period. The study flowchart is shown in Figure 3.

Baseline clinical, echocardiographic, laboratory characteristics, and medical treatments according to ICD shock status are summarized in Table 1. There were no significant differences between the groups in terms of age, sex, body mass index, HF etiology, or baseline medical therapy (*p* > 0.05 for all). According to NYHA functional class, patients in the ICD shock group were more symptomatic, with a significantly higher proportion of NYHA class II and III patients (*p* < 0.001).

The ICD shock group had a significantly lower median LVEF compared with the non-shock group (28.0% [24.0–35.0] vs. 33.5% [28.3–37.8], *p* = 0.009). Patients who experienced ICD shocks also had significantly higher NT-proBNP levels and lower serum potassium and adjusted calcium levels compared with those without shocks (*p* < 0.001, *p* = 0.016, and *p* = 0.012, respectively).

Baseline ECG characteristics and the distribution of abnormal ECG markers are presented in Table 2. Heart rate did not differ significantly between the groups (*p* = 0.238). In contrast, patients who experienced appropriate ICD shocks had significantly longer QRS duration and QTc interval than those without shocks (both *p* < 0.001).

**Figure 4:**
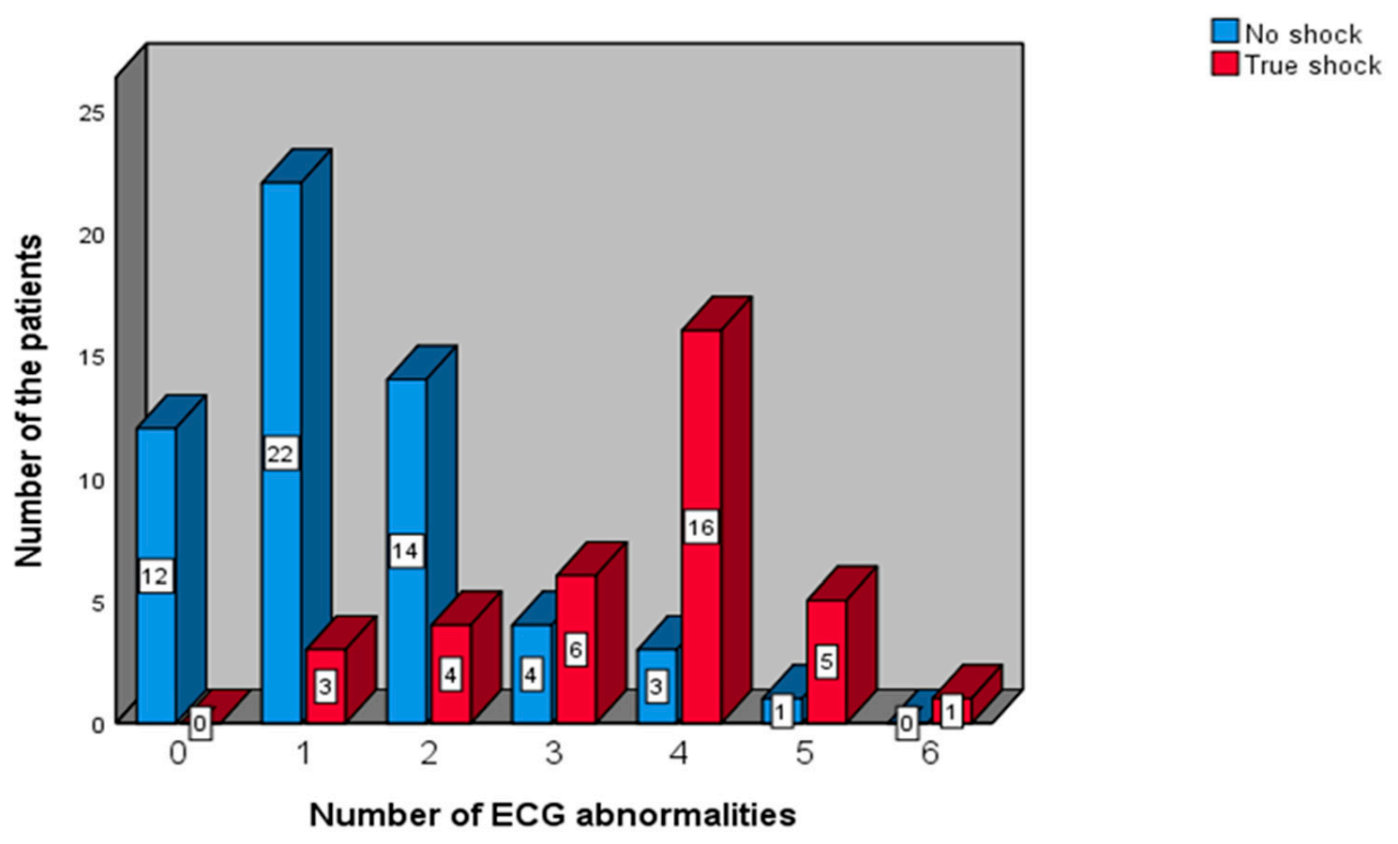
Distribution of the number of shocked and non-shocked patients according to the number of electrocardiographic abnormalities.

Consistent with these findings, the median ERS was markedly higher in the ICD shock group than in the non-shock group (4.0 [3.0–5.0] vs. 1.0 [1.0–2.0], *p* < 0.001). All abnormal ECG markers included in the ERS, except the electrocardiographic LVH marker, were significantly more prevalent in patients who received ICD shocks. The distribution of ERS values in the study population is illustrated in Figure 4. Although all-cause mortality did not differ between the groups (*p* = 0.252), patients who experienced ICD shocks had a significantly higher rate of HF-related hospitalizations within one year (*p* = 0.014).

**Figure 5:**
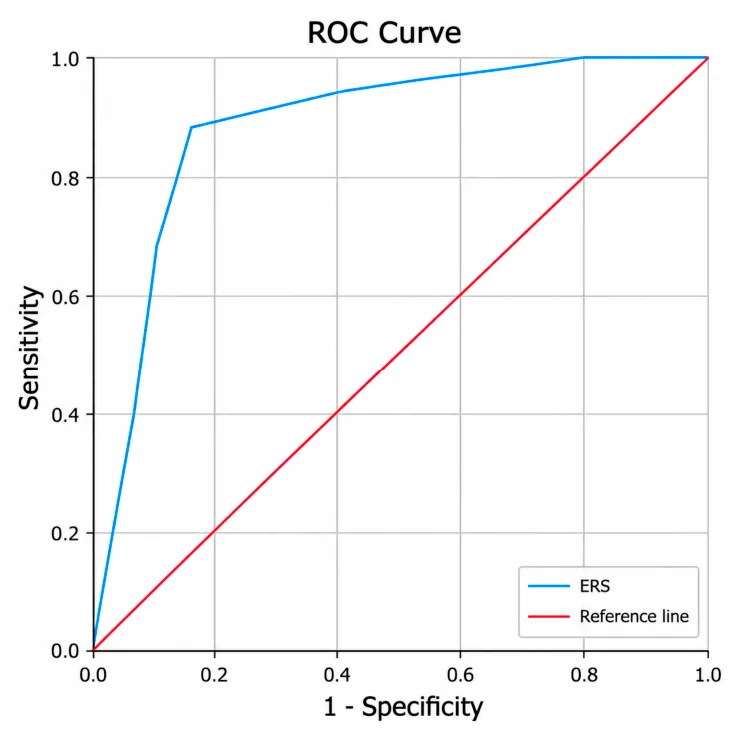
The ROC curve evaluating the discriminative ability of the electrical risk score for appropriate ICD shocks.

The ROC curve analysis demonstrated that the ERS had strong discriminative ability for predicting appropriate ICD shocks. An ERS cut-off value of 2.5 predicted ICD shocks with a sensitivity of 88.6% and specificity of 83.9% (AUC 0.882, 95% CI 0.797–0.941, *p* < 0.001) (Figure 5). Based on this threshold, patients were categorized as ERS < 3 (n = 55) or ERS ≥ 3 (n = 36), and their clinical and ECG characteristics are summarized in Table 3.

Patients with ERS ≥ 3 had significantly higher rates of anti-tachycardia pacing therapy and heart failure-related hospitalizations compared with those with ERS < 3 (*p* < 0.001 and *p* = 0.004, respectively), whereas all-cause mortality was similar between the groups. Patients with ERS ≥ 3 also had significantly longer QRS duration and QTc interval, while heart rate and LVEF did not differ significantly between ERS categories.

In the multivariable logistic regression model adjusted for age, LVEF, and NT-proBNP, ERS remained independently associated with appropriate ICD shocks (aOR 2.721, 95% CI 1.801–4.112, *p* < 0.001). Age, LVEF, and NT-proBNP were not independently associated with appropriate ICD shocks in the adjusted model (Table 4).

**Figure 6:**
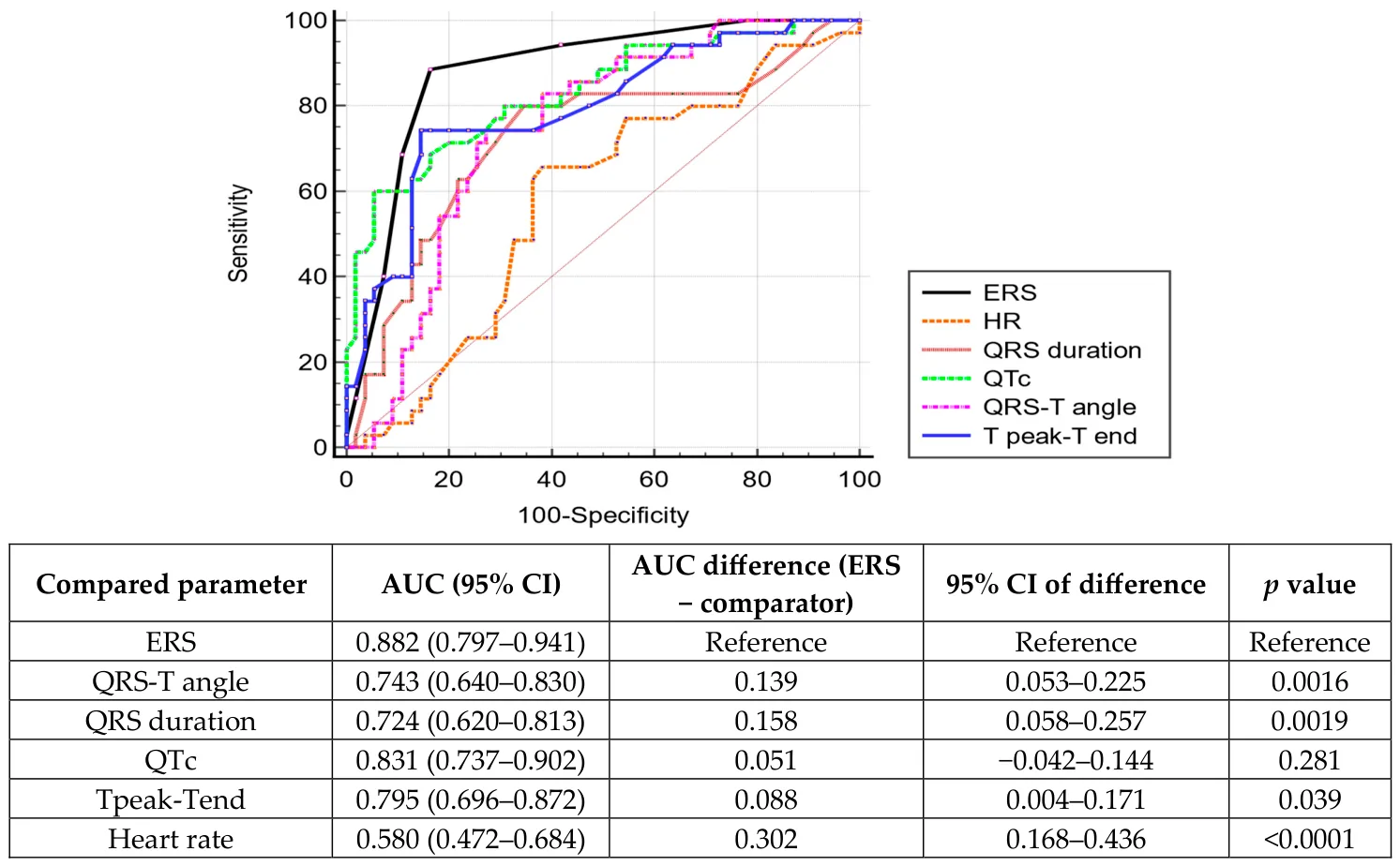
DeLong test analysis results and pairwise comparison of AUCs. AUC, area under curve; CI, confidence interval; ERS, electrical risk score; QTc, corrected QT.

In pairwise ROC curve comparisons using the DeLong test, ERS demonstrated the highest discriminative performance for predicting ICD shocks (AUC 0.882), significantly outperforming frontal QRS T angle, QRS duration, Tp-e interval, and heart rate. The difference between ERS and QTc interval was not statistically significant (Figure 6).

## 4. Discussion

In this study, we evaluated the association between the ERS and malignant ventricular tachyarrhythmia-related ICD shocks in patients with HF who underwent ICD implantation for primary prevention. Consistent with a more advanced HF phenotype, patients who experienced appropriate ICD shocks were more symptomatic and had lower LVEF and higher NT-proBNP levels. Electrocardiographic markers reflecting abnormalities in conduction, depolarization, and repolarization were more prevalent in this group, and the ERS remained independently associated with appropriate ICD shocks after adjustment for age, LVEF, and NT-proBNP. The ERS demonstrated high discriminative ability, outperforming most individual ECG parameters.

In the one-year follow-up period, ERS showed a strong association with appropriate ICD shocks. The ROC curve analysis demonstrated that an ERS threshold of 3 provided high sensitivity and specificity for predicting appropriate ICD shocks. Approximately 80% of patients with ERS ≥ 3 experienced an appropriate ICD shock, whereas nearly 85% of patients without shocks had ERS values below this threshold. Patients with ERS ≥ 3 also experienced higher rates of anti-tachycardia pacing and HF-related hospitalizations. Consistently, pairwise ROC comparisons using the DeLong test showed that ERS had the highest discriminative performance among the evaluated ECG parameters. These findings suggest that ERS captures a broader arrhythmogenic substrate than single ECG markers.

The ERS consists of seven ECG parameters, each reflecting distinct but complementary aspects of myocardial electrical and structural remodeling. Parameters included in the score represent autonomic regulation, ventricular depolarization and repolarization abnormalities, and structural remodeling, all of which are well-established contributors to ventricular arrhythmogenesis in HF. Prior studies have demonstrated that increased resting heart rate is associated with adverse outcomes in HF [13,21]. Widened QRS-T angle reflects heterogeneity of ventricular depolarization and repolarization and has been shown to predict appropriate ICD therapy [12,22,23]. Prolonged Tp-e interval and QTc interval as a result of ventricular damage, abnormal potassium channel function and reduced repolarization reserve have been shown in many studies to be predictors of ventricular arrhythmia and have been recommended for risk stratification of patients with HF [24,25]. Prolonged QRS duration indicates intraventricular conduction delay and advanced myocardial dysfunction, which are associated with sudden cardiac death [26,27]. Delayed QRS transition zone has been linked to adverse left ventricular remodeling and arrhythmic risk [15], while electrocardiographic LVH reflects complex structural remodeling that predisposes patients to malignant ventricular arrhythmias [28]. Although each of these parameters individually conveys prognostic information, their integration into a composite score provides a more comprehensive assessment of the underlying arrhythmogenic substrate. In this context, ERS demonstrated superior predictive performance compared with most individual ECG parameters, with the exception of QTc. The lack of a significant difference between ERS and QTc in the DeLong analysis is not unexpected, as prolonged QTc is a well-established marker of myocardial disease severity and arrhythmic risk [25,26].

Patients who experienced appropriate ICD shocks had lower LVEF and higher NT-proBNP levels, indicating more advanced HF. However, neither LVEF nor NT-proBNP remained independently associated with appropriate ICD shocks in the multivariable analysis. This finding is consistent with previous studies showing that LVEF, although an established prognostic marker in HF, does not reliably identify patients at increased risk of ventricular arrhythmias requiring ICD therapy [29]. These conventional markers of HF severity may, therefore, not fully reflect the underlying arrhythmogenic substrate. In contrast, ERS remained independently associated with appropriate ICD shocks after adjustment for age, LVEF, and NT-proBNP, suggesting that ERS may provide complementary information for arrhythmic risk stratification beyond conventional measures of HF severity.

Previous studies have explored the prognostic value of ERS in different clinical settings. Aro et al. initially identified ERS ≥ 4 as a high risk threshold for sudden cardiac death [15]. However, Elmas et al. accepted the best cut-off value of ERS as 3 because ERS > 4 represented a very small group in other studies and ERS ≥ 3 predicted mortality in their study [16]. In view of the data, the ERS cut-off value for predicting ventricular arrhythmia was determined to be ≥2.5 in ROC curve analysis, in our study. The study population was divided into two groups, ERS ≥ 3 and ERS < 3. In addition to the primary endpoint of appropriate ICD shock, ATP-terminated ventricular tachycardia was evaluated separately as a secondary device-related outcome. The treatment with ATP was found to be significantly higher in HF patients with ERS ≥ 3. We also found that poor outcomes and hospitalization were more common in patients with ERS ≥ 3. The assumption was made that these results were consistent due to the known fact that patients suffering from shock have more advanced HF. The absence of a significant difference in mortality between the two groups can be attributed to the limited study population and the short follow-up period.

A standard 12-lead ECG is routinely obtained during outpatient follow-up of patients with HF. The ERS can be easily calculated from a 12-lead ECG, making it a simple, accessible, and cost-effective tool. In patients with symptomatic and advanced HF undergoing ICD follow-up, ERS may, therefore, be readily assessed as part of routine clinical evaluation. Previous studies have demonstrated that ICD shocks may adversely affect quality of life and are associated with an increased risk of mortality [5,6]. In this context, ERS may provide additional information during routine clinical assessment and may help identify patients who could benefit from closer and more individualized follow-up. However, whether ERS can be used to guide specific therapeutic or device-programming decisions requires further prospective investigation.

## 5. Limitations

This study has several limitations. Its retrospective and single-center design and the modest sample size may limit generalizability. The ERS was calculated from a single ECG obtained during routine follow-up, and dynamic changes in ERS over time could not be assessed. The follow-up period was limited to one year, which may have reduced the ability to detect long-term differences in mortality and adverse outcomes. As the ERS cut-off was derived from the present cohort, further validation in larger and independent populations would strengthen the generalizability of the proposed ERS ≥ 3 threshold. Additionally, the exact time to the first appropriate ICD shock was not systematically available, precluding time-to-event analyses. Detailed episode-level device data, including VT/VF cycle lengths and therapy sequences, were not consistently available for all patients. Although ischemic and non-ischemic etiologies were considered, the relationship between ERS and myocardial scar burden or genetically mediated arrhythmic syndromes could not be evaluated, as cardiac magnetic resonance imaging data were not systematically available.

## 6. Conclusions

In patients with HF undergoing ICD implantation for primary prevention, higher ERS was independently associated with appropriate ICD shocks during one-year follow-up. As a simple ECG-derived measure, ERS may provide additional information for risk assessment and support a more individualized approach to clinical follow-up. Further studies in larger and independent cohorts are warranted to validate these findings and clarify the potential clinical role of ERS.

## Figures and Tables

**Table 1:** Comparison of variables between groups with and without ICD shock.

Variables	Without Shock Group (*n* = 56)	Shock Group (*n* = 35)	*p* Value
Age, years	50.2 ± 14.3	54.9 ± 16.9	0.177
Male, *n* (%)	27 (48.2)	20 (57.1)	0.407
Body mass index, kg/m^2^	27.6 [23.7–31.2]	26.8 [24.9–30.6]	0.970
Dyslipidemia, *n* (%)	35 (62.5)	20 (57.1)	0.611
Hypertension, *n* (%)	28 (50.0)	22 (62.9)	0.230
Diabetes mellitus, *n* (%)	17 (30.4)	15 (26.8)	0.224
CVD, *n* (%)	4 (7.1)	4 (11.4)	0.482
Echocardiographic characteristics			
EDD, mm	62.2 ± 8.9	62.2 ± 7.4	0.913
LA, mm	47.8 ± 5.9	45.9 ± 5.7	0.136
LVEF, %	33.5 [28.3–37.8]	28.0 [24.0–35.0]	0.009
HF etiology			
Non-ischemic CMP, *n* (%)	19 (33.9)	10 (28.6)	0.594
Ischemic CMP, *n* (%)	37 (66.0)	25 (71.4)	
NYHA class			
NYHA class I, *n* (%)	32 (57.1)	1 (2.9)	<0.001
NYHA class II, *n* (%)	23 (41.0)	24 (68.6)	
NYHA class III, *n* (%)	1 (1.9)	10 (28.6)	
Medication			
Statin, *n* (%)	27 (48.2)	16 (45.7)	0.816
Beta blocker, *n* (%)	55 (98.2)	35 (97.2)	0.998
ACEi/ARB/ARNI, *n* (%)	49 (87.5)	27 (77.1)	0.195
MRA, *n* (%)	48 (85.7)	24 (68.6)	0.050
CCB, *n* (%)	6 (10.7)	7 (20.0)	0.218
Digoxin, *n* (%)	10 (17.9)	10 (28.6)	0.230
Ivabradine, *n* (%)	12 (21.4)	4 (11.4)	0.223
Amiodarone, *n* (%)	19(33.9)	13(37.1)	0.755
SGLT2i, *n* (%)	23(41.1)	16(45.7)	0.663
Laboratory parameters			
NT-proBNP, pg/mL	356.0 [182.0–771.0]	753.0 [480.0–2254.0]	<0.001
eGFR, mL/min/1.73 m^2^	84.5 ± 24.8	73.7 ± 26.7	0.059
Urea, mg/dL	45.5 [32.3–61.2]	45.0 [35.0–53.0]	0.822
Creatinine, mg/dL	1.0 [0.9–1.2]	1.0 [0.9–1.2]	0.428
Sodium, mEq/L	138.0 [136.0–140.0]	139.0 [137.0–141.0]	0.208
Potassium, mEq/L	4.4 [4.2–4.8]	4.1 [3.8–4.4]	0.016
Magnesium, mEq/L	2.1 [1.9–2.3]	2.0 [1.7–2.2]	0.115
Adjusted calcium, mEq/L a	9.2 ± 0.7	8.7 ± 0.9	0.012
Albumin, mg/dL	4.3 [3.8–4.6]	4.2 [3.9–4.4]	0.523

a Adjusted calcium values are corrected according to albumin levels. Abbreviations: ACEi, angiotensin-converting enzyme inhibitor; ARB, angiotensin receptor blocker; ARNI, angiotensin receptor neprilysin inhibitor; CCB, calcium channel blocker; CMP, cardiomyopathy; CVD, cerebrovascular disease; EDD, end diastolic diameter; eGFR, estimated glomerular filtration rate; HF, heart failure; LA, left atrium; LVEF, left ventricular ejection fraction; MRA, mineralocorticoid receptor antagonist; NT-proBNP, N-terminal pro-B-type natriuretic peptide; NYHA, New York Heart Association; SGLT2i, Sodium-glucose cotransporter 2 inhibitor.

**Table 2:** Comparison of basic electrocardiographic variables, clinical outcomes and distribution of electrographic abnormal markers between ICD shock and non-shock groups.

Variables	Without Shock Group (*n* = 56)	Shock Group (*n* = 35)	*p* Value
Baseline ECG			
Heart rate, bpm	71.0 [63.0–81.8]	75.0 [69.0–82.0]	0.238
QRS duration, ms	90.0 [85.0–102.0]	107.0 [96.0–150.0]	<0.001
QTc, ms	416.3 ± 29.9	459 ± 36.6	<0.001
Electrical risk score	1.0 [1.0–2.0]	4.0 [3.0–5.0]	<0.001
Electrocardiographic abnormal markers			
Heart rate > 75, *n* (%)	22 (39.2)	21 (60.0)	0.005
QRS > 110 ms, *n* (%)	8 (14.2)	18 (51.4)	<0.001
QTc Bazett > 450 or >460 ms, *n* (%)	6 (10.7)	21 (60.0)	<0.001
Tpeak–Tend > 89 ms, *n* (%)	9 (16.0)	26 (74.3)	<0.001
QRS-T angle > 90°, *n* (%)	14 (25.0)	23 (65.7)	<0.001
QRS transition zone ≥ V4, *n* (%)	18 (32.1)	24 (68.6)	0.001
Electrocardiographic left ventricular hypertrophy, *n* (%)	10 (17.9)	9 (25.7)	0.393
Exitus, *n* (%)	3 (5.4)	5 (14.2)	0.252
Hospitalization, *n* (%)	16 (28.6)	19 (54.3)	0.014

**Abbreviations:** QTc, corrected QT; V4, 4th precordial lead.

**Table 3:** Comparison of electrocardiographic characteristics and clinical outcomes of patients with ERS < 3 and ≥3.

Variables	ERS < 3 (*n* = 55)	ERS ≥ 3 (*n* = 36)	*p* Value
Exitus, *n* (%)	2 (4.0)	6 (16.6)	0.132
ATP, *n* (%)	10 (18.2)	32 (88.8)	<0.001
Hospitalization, *n* (%)	13 (23.6)	22 (61.1)	0.004
LVEF, %	32.5 ± 7.7	30.1 ± 8.5	0.182
Heart rate, bpm	72.3 ± 12.5	74.0 ± 12.0	0.508
QRS duration, ms	88.0 [84.0–96.0]	118.0 [100.5–158.5]	<0.001
QTc, ms	411.5 [391.8–439]	465.0 [437.0–477.5]	<0.001
Tpeak–Tend, ms	79.0 [68.0–83.3]	97.5 [90.2–111.2]	<0.001
QRS-T angle, °	28.5 [9.8–62.8]	117.5 [85.5–154.5]	<0.001
QRS transition zone ≥ V4, *n* (%)	13 (23.6)	29 (80.5)	<0.001
Electrocardiographic left ventricular hypertrophy, *n* (%)	9 (16.3)	10 (27.8)	0.419

**Abbreviations:** ATP, anti-tachycardia pacing; LVEF, left ventricular ejection fraction; QTc, corrected QT; V4, 4th precordial lead.

**Table 4:** Multiple logistic regression analysis identifying independent predictors appropriate for ICD shocks.

Variables *	OR (95% CI)	*p* Value	aOR (95% CI)	*p* Value
Age	1.020(0.992–1.050)	0.159	0.995(0.956–1.035)	0.809
NT-proBNP	1.042(1.005–1.081)	0.026	1.025(0.980–1.073)	0.284
LVEF	0.954(0.902–1.010)	0.104	0.963(0.894–1.037)	0.319
ERS	2.812(1.896–4.172)	<0.001	2.721(1.801–4.112)	<0.001

* Age, per year; NT-proBNP, per100 pg/mL; LVEF, per 1% increase; ERS, per 1-point increase. **Abbreviations:** aOR, adjusted odds ratio; CI, confidence interval; ERS, electrical risk score; LVEF, left ventricular ejection fraction; NT-proBNP, N-terminal pro-B-type natriuretic peptide; OR, odds ratio.

## Data Availability

The datasets analyzed during the current study are available from the corresponding author on reasonable request, subject to institutional and ethical restrictions.
